# Impacts of age and sex on retinal layer thicknesses measured by spectral domain optical coherence tomography with Spectralis

**DOI:** 10.1371/journal.pone.0194169

**Published:** 2018-03-09

**Authors:** María Nieves-Moreno, José M. Martínez-de-la-Casa, Laura Morales-Fernández, Rubén Sánchez-Jean, Federico Sáenz-Francés, Julián García-Feijoó

**Affiliations:** Ophthalmology Unit, Hospital Clínico San Carlos, Department of Ophthalmology, Faculty of Medicine, Universidad Complutense de Madrid, and Instituto de Investigación Sanitaria del Hospital Clínico San Carlos, Madrid, Spain; University of Florida, UNITED STATES

## Abstract

**Objective:**

To examine differences in individual retinal layer thicknesses measured by spectral domain optical coherence tomography (SD-OCT) (Spectralis^®^) produced with age and according to sex.

**Design:**

Cross-sectional, observational study.

**Methods:**

The study was conducted in 297 eyes of 297 healthy subjects aged 18 to 87 years. In one randomly selected eye of each participant the volume and mean thicknesses of the different macular layers were measured by SD-OCT using the instrument's macular segmentation software.

**Main outcome measures:**

Volume and mean thickness of macular retinal nerve fiber layer (mRNFL), ganglion cell layer (GCL), inner plexiform layer (IPL), inner nuclear layer (INL), outer plexiform layer (OPL), outer nuclear layer (ONL), retinal pigmentary epithelium (RPE) and photoreceptor layer (PR).

**Results:**

Retinal thickness was reduced by 0.24 μm for every one year of age. Age adjusted linear regression analysis revealed mean GCL, IPL, ONL and PR thickness reductions and a mean OPL thickness increase with age. Women had significantly lower mean GCL, IPL, INL, ONL and PR thicknesses and volumes and a significantly greater mRNFL volume than men.

**Conclusion:**

The thickness of most retinal layers varies both with age and according to sex. Longitudinal studies are needed to determine the rate of layer thinning produced with age.

## Introduction

New optical coherence tomography (OCT) techniques are excellent tools for the noninvasive, in vivo, high-resolution visualization of the retina. Such tools can be used to monitor retinal thickness in the region of the macula as this measurement is useful for the diagnosis and follow up of many macular and optic nerve disorders. It is, therefore, important that we can distinguish between pathological changes and those associated with age. In effect, data for the subfields defined in the Early Treatment Diabetic Retinopathy Study (ETDRS) have shown that retinal thickness decreases with age when measured at both the pericentral and peripheral ring of the macula.[1–5]

Using the Spectralis OCT (Heidelberg Engineering) it is possible to automatically measure overall retinal thickness and the thickness of each individual retinal layer with good reproducibility.[6] The objective of this study was to examine the impacts of age and sex on each of the 9 macular retinal layers automatically segmented by the Spectralis OCT software. To the best of our knowledge, this is the first study to provide normality data for the effects of age on each retinal layer with Spectralis-OCT.

## Methods

This study was designed as an observational, cross-sectional analysis of 300 eyes of 300 healthy volunteers. The study protocol was approved by the Institutional Review Board of the Hospital Clínico San Carlos, Madrid, Spain, and followed Helsinki declaration guidelines. Participant selection was in line with the recommendations of Realini et al.[7]

### Participants

We recruited 300 healthy white subjects with no eye symptoms among the patients and their relatives visiting our clinic for a routine ophthalmologic examination, and also among the hospital staff. All participants were > 18 years old. All subjects signed an informed consent form after the study protocol and its purpose were explained to them in detail. Participants first underwent a complete ophthalmic examination, including, biomicroscopy, intraocular pressure (IOP) measurement, fundus test, and refraction. The medical records of the individuals were also examined. For each patient, one eye was randomly selected for the final analysis.

Every eye included in our study had a vision equal to or better than 20/40, a sphere between +/-5 diopters and a cylinder between +/-3 diopters. Subjects recently undergoing cataract surgery (<1 year) or with a medical history of IOP >21 mmHg, glaucoma, or a macular, neuro-ophthalmological or retinal disease were excluded. Further exclusion criteria were a history of neurological disease or any uncontrolled disease.

### Optical coherence tomography

An SD-OCT macular examination was performed without pupil dilation using the Spectralis OCT (Heidelberg Engineering, Heidelberg, Germany) in a dark room in each participant on the same day. The scan was conducted on a 20×20 degree cube with 49 raster lines separated by 120 μm, each containing 1046 pixels. This instrument's automatic eye tracking technology maintains fixation on the retina. Only well-centered, well-segmented images with a signal strength of >20db were used for analysis. All scans were performed and checked by the same experienced ophthalmologist and no manual adjustments to retinal layer segmentation were made.

Macular and inner retinal layer thicknesses were measured on 1, 3 and 6 mm rings as indicated in the ETDRS macular map. The variables recorded for each eye or participant were the means of each set of EDRS subfield thicknesses recorded for each retinal layer and the volumes of each of these layers. The 1 mm rings was named central circle, the 3 mm ring was named inner circle and the 6 mm ring was named outer circle. Volumes are automatically calculated by Spectralis OCT.

**Fig 1:**
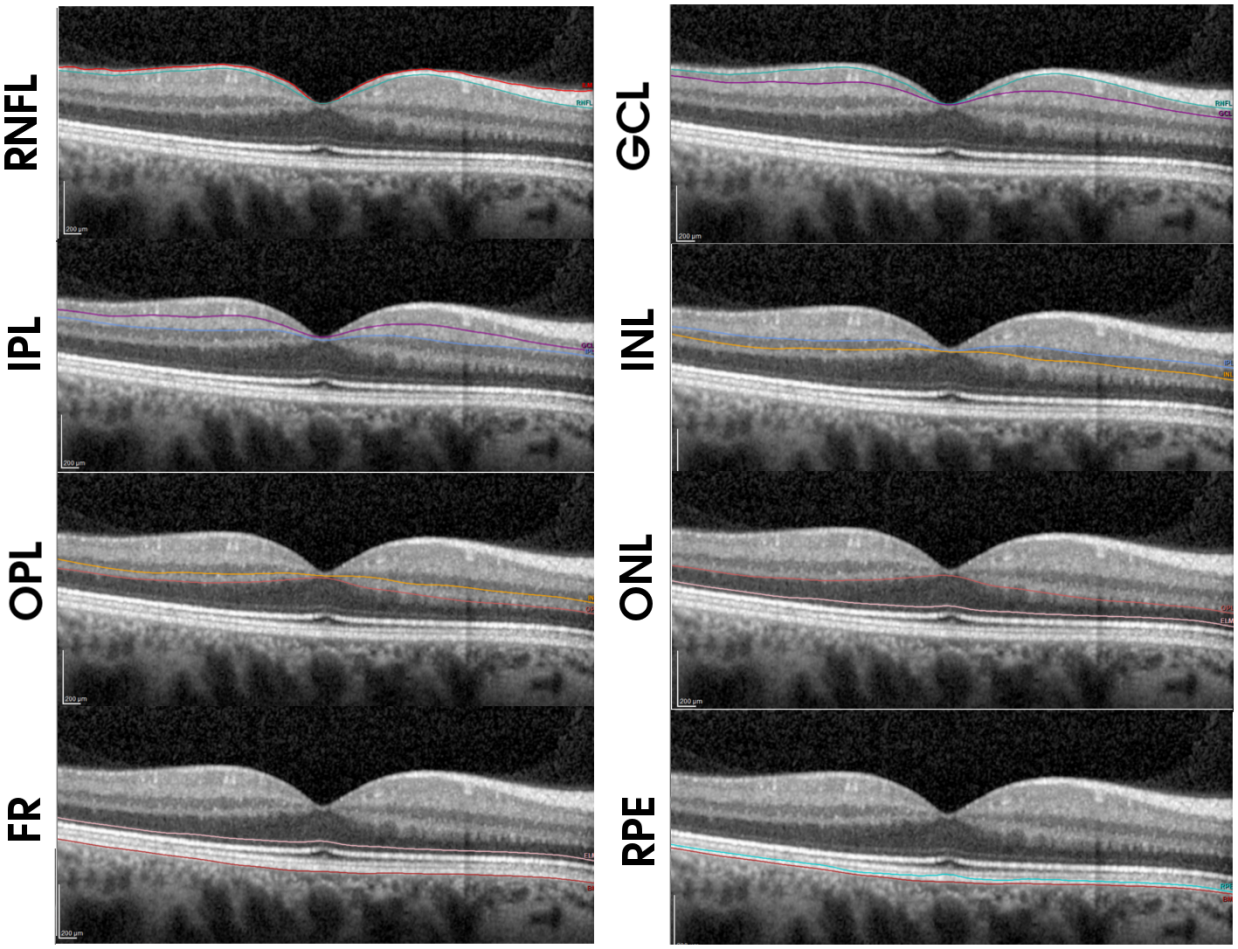
Spectralis segmentation of the retinal nerve fiber layer (mRNFL), ganglion cell layer (GCL), inner plexiform layer (IPL), inner nuclear layer (INL), outer plexiform layer (OPL), outer nuclear layer (ONL), retinal pigmentary epithelium (RPE) and photoreceptor layer (PR).

Using the new Spectralis segmentation software (6.0c version), the following measurements were acquired in each subject: overall retinal thickness, and thicknesses of the macular retinal nerve fiber layer (mRNFL), ganglion cell layer (GCL), inner plexiform layer (IPL), inner nuclear layer (INL), outer plexiform layer (OPL), outer nuclear layer (ONL—including Henle's fiber layer), retinal pigmentary epithelium (RPE) and photoreceptor layer (PR—being this layer the thickness between the outer limiting membrane and the Burch membrane) (Fig 1). All scans were performed by the same experienced operator and no manual adjustments to retinal layer segmentation were made.

### Statistical analysis

Data were obtained for one randomly selected eye of each participant. Descriptive statistics are reported as the mean, range and standard deviation. All p values were adjusted using the Bonferroni factor. Correlation between different measurements was defined through Pearson correlation coefficients. The effects of age and sex were analyzed by simple regression analysis. All statistical tests were performed using the SPSS software package version 15 (SPSS Inc., Chicago, IL, USA). Significance was set at p < 0.05.

## Results

Of the 300 subjects recruited, 3 were excluded because of artifacts in the macula images. This left a study population of 297 subjects: 179 women and 118 men of mean age 56.07 ± 18.72 years. In these subjects, data were obtained for 150 right eyes and 147 left eyes. Mean ages were 55.70 ± 19.21 for the men and 56.64 ± 18.02 for the women, with no significant difference between these ages (p = 0.34).

**Fig 2:**
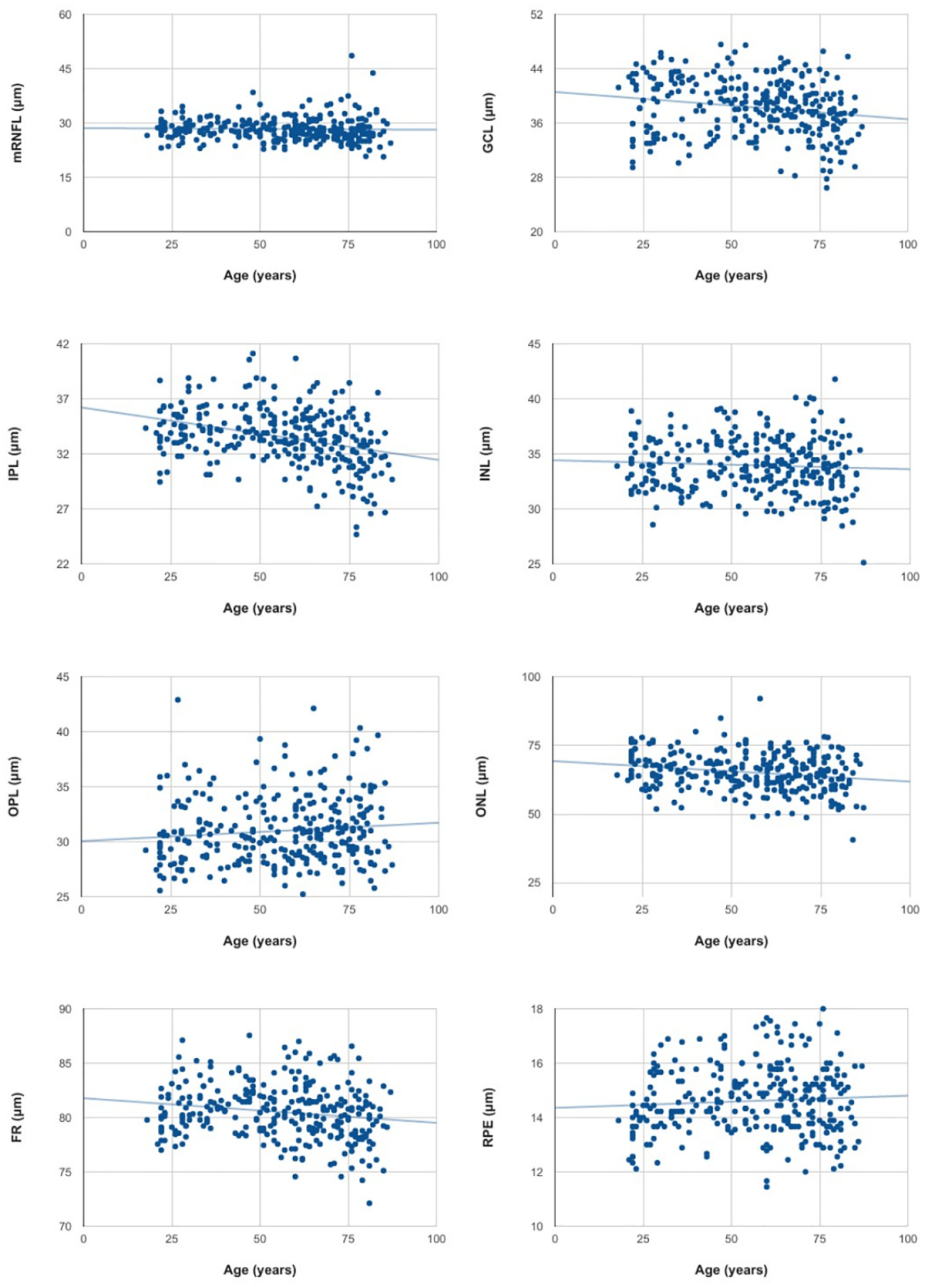
Scatterplots of simple linear regression between age and mean mRNFL, GCL, IPL, INL, OPL, ONL, PR and RPE thickness recorded in 297 subjects.

For each retinal layer, mean thickness was calculated as the arithmetic mean of the thicknesses of all nine ETDRS subfields. The results of the age adjusted linear regression analysis of the mean thickness and volume of each retinal layer are shown in Tables 1 and 2 and Fig 2. This analysis revealed thinning of GCL, IPL, ONL and PR and thickening of OPL with age. No significant differences with age were observed in the remaining retinal layers.

**Table 1:** Simple linear regression between age and mean retinal layer thickness and volume for the study population of 297 subjects.

		Mean (+/- SD)	R	Loss μm*year (SE)	p
**Retina**	Mean thickness (μm)	312.26 (+/- 12.97)	**-0.35**	-0.240 (0.042)	<**0.001**
	Volume (mm^3^)	8.59 (+/- 0.36)	**-0.35**	-0.008 (0.001)	<**0.001**
**mRNFL**	Mean thickness (μm)	28.33 (+/- 3.20)	-0.03	0.012 (0.01)	0.195
	Volume (mm^3^)	0.96 (+/- 0.12)	-0.01	0.001 (0.001)	0.213
**GCL**	Mean thickness (μm)	38.31 (+/- 4.14)	-0.18	-0.051 (0.13)	<**0.001**
	Volume (mm^3^)	1.02 (+/- 0.11)	-0.03	-0.001 (0.001)	**0.003**
**IPL**	Mean thickness (μm)	33.54 (+/- 2.76)	**-0.34**	-0.054 (0.008)	<**0.001**
	Volume (mm^3^)	0.88 (+/- 0.07)	**-0.32**	-0.001 (0.001)	<**0.001**
**INL**	Mean thickness (μm)	33.96 (+/- 2.42)	-0.20	-0.015 (0.008)	0.059
	Volume (mm^3^)	0.94 (+/- 0.06)	-0.06	-0.001 (0.001)	<**0.001**
**OPL**	Mean thickness (μm)	30.99 (+/- 3.03)	0.10	0.022 (0.010)	**0,029**
	Volume (mm^3^)	0.83 (+/- 0.07)	0.13	0.001 (0.001)	**0,015**
**ONL**	Mean thickness (μm)	65.12 (+/- 6.82)	-0.21	-0.098 (0.22)	<**0.001**
	Volume (mm^3^)	1.67 (+/- 0.18)	-0.29	-0.003 (0.001)	<**0.001**
**PR**	Mean thickness (μm)	80.50 (+/- 2.50)	-0.17	-0.022 (0.008)	**0,008**
	Volume (mm^3^)	2.23 (+/- 0.07)	-0.14	<0.001	**0.047**
**RPE**	Mean thickness (μm)	14.61 (+/- 1.23)	0.07	0.005 (0.004)	0.233
	Volume (mm^3^)	0.39 (+/- 0.32)	0.09	<0.001	0.087

Note that mean thicknesses represent the means for all nine EDRS subfields.

**Table 2:** Simple linear regression between age and retinal layer thickness of the central, inner and outer circle for the study population of 297 subjects.

		Mean (+/- SD) (μm)	R	Loss μm*year (SE)	p
**Retina**	Central circle	278.20 (+/- 21.13)	-0.03	-0.034 (0.070)	0.664
	Inner circle	338.38 (+/- 17.70)	**-0.32**	-0.250 (0.43)	<**0.001**
	Outer circle	294.64 (+/- 13.05)	**-0.32**	-0.225 (0.038)	<**0.001**
**mRNFL**	Central circle	12.71 (+/- 2.29)	0.24	0.008 (0.007)	0,301
	Inner circle	22.65 (+/- 2.27)	-0.06	-0.007 (0.007)	0.333
	Outer circle	37.90 (+/- 5.34)	-0.01	-0.004 (0.017)	0.806
**GCL**	Central circle	17.63 (+/- 5.03)	-0.28	-0.056 (0.016)	<**0.001**
	Inner circle	48.35 (+/- 5.81)	-0.19	-0.058 (0.018)	**0.001**
	Outer circle	33.44 (+/- 4,03)	-0.08	-0.016 (0.013)	0.195
**IPL**	Central circle	22.05 (+/- 3.67)	-0.28	-0.011 (0.012)	0.362
	Inner circle	41.31 (+/- 3.39)	**-0.38**	-0.069 (0.010)	<**0.001**
	Outer circle	28.64 (+/- 2.54)	-0.25	-0.034 (0.008)	<**0.001**
**INL**	Central circle	20.52 (+/- 5.05)	**0.37**	0.060 (0.015)	<**0.001**
	Inner circle	39.23 (+/- 3.20)	0.00	8.59 ^e^-0.005^	0.993
	Outer circle	32.05 (+/- 2.25)	-0.28	-0.034 (0.007)	<**0.001**
**OPL**	Central circle	26.38 (+/- 5.50)	0.18	0.004 (0.018)	0.817
	Inner circle	35.18 (+/- 4.46)	0.09	0.022 (0.014)	0.109
	Outer circle	27.94 (+/- 2.18)	0.15	0.018 (0.007)	**0.008**
**ONL**	Central circle	92.71 (+/- 10.14)	0.17	0.017 (0.033)	0.598
	Inner circle	68.41 (+/- 8.08)	-0.14	-0.061 (0.025)	**0.015**
	Outer circle	54.93 (+/- 6.16)	-0.35	-0.114 (0.018)	<**0.001**
**PR**	Central circle	88.12 (+/- 4.30)	**-0.30**	-0.073 (0.013)	<**0.001**
	Inner circle	80.98 (+/- 2.73)	-0.13	-0.019 (0.008)	**0.022**
	Outer circle	78.12 (+/- 2.37)	-0.12	-0.015 (0.007)	**0.039**
**RPE**	Central circle	16.90 (+/- 2.16)	0.15	-0.015 (0.007)	**0.033**
	Inner circle	15.23 (+/- 1.49)	0.10	0.008 (0.005)	0.097
	Outer circle	13.41 (+/- 1.21)	0.09	0.005 (0.003)	0.135

The thinning of the whole retina detected was a 0.24 μm thickness reduction for every year of participant age. The retinal layer that showed most thinning with age was ONL, which experienced a thickness reduction of 0.098 μm/year.

Table 3 shows the differences observed between sexes in the linear regression analysis. Overall retinal thickness varied between both sexes by 7.113 μm. In women, GCL, IPL, INL, ONL and PR thicknesses and volumes were significantly reduced compared to the measurements recorded in men. The only remaining significant difference noted was a greater mRNFL volume in women than men despite no mean mRNFL thickness difference.

**Table 3:** Simple linear regression between age and mean retinal layer thickness and volume for the study population of 297 subjects.

		Difference men vs women (SE)	p
**Retina**	Mean thickness (μm)	7.113 (1.516)	<**0.001**
	Volume (mm^3^)	0.151 (0.42)	<**0.001**
**mRNFL**	Mean thickness (μm)	-0.508 (0.391)	0.195
	Volume (mm^3^)	-0.030 (0.015)	**0.044**
**GCL**	Mean thickness (μm)	1.609 (0.499)	**0,001**
	Volume (mm^3^)	0.044 (0.014)	**0.002**
**IPL**	Mean thickness (μm)	0.837 (0.308)	**0.007**
	Volume (mm^3^)	0.019 (0.008)	**0.019**
**INL**	Mean thickness (μm)	1.122 (0.302)	<**0.001**
	Volume (mm^3^)	0.022 (0.008)	**0.004**
**OPL**	Mean thickness (μm)	0.016 (0.385)	0.967
	Volume (mm^3^)	0.006 (0.009)	0.527
**ONL**	Mean thickness (μm)	3.772 (0.826)	<**0.001**
	Volume (mm^3^)	0.097 (0.021)	<**0.001**
**PR**	Mean thickness (μm)	0.704 (0.313)	**0.025**
	Volume (mm^3^)	0.021 (0.008)	**0.012**
**RPE**	Mean thickness (μm)	0.215 (0.158)	0.173
	Volume (mm^3^)	0.004 (0.004)	0.297

## Discussion

Based on the findings of other OCT studies revealing negative correlation between age and overall retinal thickness,[1–5] this study sought to examine the effects of age and sex on the thicknesses of- the individual retinal layers as measured by the Spectralis OCT segmentation tool.

### Retinal layer thickness changes associated with age

The overall macular thinning observed here with age is consistent with the findings of studies including those of Kanai et al.,[2] Manassalorn et al.,[3] and Appukuttan et al.[4] in which negative correlation was detected between every retinal ETDRS zone but the central zone and age. In contrast, Grover et al.[8] found no significant differences with age perhaps because of the small number of subjects examined (n = 50).

As we age, neurons in the inner retinal layers are lost.[9] According to histological findings, between 0.3 and 0.6% of neurons are lost every year.[10] Ooto et al.[11] reported a linear decrease with age of -0.07 and -0.05 μm per year in GCL and IPL thickness, respectively, as measured using the Topcon 3D OCT (OCT- 1000; Topcon, Tokyo, Japan) in 256 healthy volunteers. In the present study, we observed correlation between reduced GCL and IPL thickness and age, indicating a rate of GCL and IPL thinning of -0.051 and -0.054 μm per year respectively. These figures are similar to the rates reported for other OCT studies [4, 12, 13]and are also in line with the results of histological studies [14], but in a lower rate of 0.17 and 0.16% of reduction every year in GCL and IPL thickness.

According to the rates of GCL and IPL thinning observed in our study participants, we could predict mean losses of 1.275 μm and 1.35 μm in the thickness of these two retinal layers respectively over the next 25 years. However, this estimate is only a theoretical value obtained from the linear relationship between age and thickness, and fails to consider, for example, if greater thinning is produced from a certain age onwards. Longitudinal studies are needed to confirm these rates of individual retinal nerve fiber layer thinning.

Knowledge of inner retinal layer thickness losses associated with age is important as any thickness reduction found could be the consequence of normal aging or of the progression of a disease such as glaucoma.[15]

Fewer studies have examined the effects of age on outer retinal layer thickness. In this study, we observed discrete positive correlation between age and OPL thickness, while ONL thickness was negatively correlated with age. In the study by Ooto et al.,[11] no significant correlation was found when examining both layers together. Hence, it seems that these layers should not be analyzed together as age may have an opposing effect on each one, possibly explaining the different results obtained.

Although aging has been traditionally associated with inner retinal layer thickness loss, the present results suggest ONL is the layer that undergoes most thinning with age. The outer nuclear layer of the retina is a hypo-reflective band that anatomically corresponds to the photoreceptor bodies. According to our results, we could estimate a mean thickness loss for this layer of 2.45 μm over 25 years, though again this rate needs to be confirmed in longitudinal and/or histological studies. On the other hand, in this study Henle's fiber layer was not measured individually and this may affect our results, since may be Henle's fiber layer the one decreasing with age [16].

The mean decrease in mean PR layer thickness produced in the present study with age is in line with the findings of others such as Won et al.[5] In this last study, significantly lower ETDRS peripheral and central PR thicknesses were noted when comparing subjects aged under 30 years and over 60 years, though no such difference was noted for ETDRS central ring thickness. In contrast, in our results the central circle had the strongest negative correlation with age (R = 0.30, p < 0,001).

In the retinal pigment epithelium, numerous structural changes with age have been described including an increase in the density of residual bodies, lipofuscin accumulation, drusen formation and Bruch's membrane thickening [17]. All these changes can lead to a RPE thickness increase with age when measured by SD-OCT. This increase could be real or the consequence of a reflectivity increase. The RPE thickening with age observed in our study was not significant; however, there have been reports of such an increase in RPE thickness with age.[12]

### Retinal layer thickness according to sex

In this study, overall retinal thickness was significantly greater in men than women; the mean thickness difference recorded in the central zone was 7.113 μm. The data collected are consistent with reports by Appukuttan et al.,[4] Mass-in et al.,[18] and Jonas et al.[19] of a significantly higher mean retinal thickness found in male compared to female subjects. In the Jonas et al. study, the mean difference in macular thickness between men and women was 8.04 μm (95% CI 10.6–5.5; p<0.001), similar to the difference observed here.

Won et al.[5] detected significant differences between both sexes in mRNFL, GCL and IPL thicknesses measured in the central, inner and outer rings. Unlike our findings, these authors observed significant differences between IPL inner ring and foveal thickness though their study cohort was considerably smaller than ours (n = 50).

The results of our study also identified a significantly greater mRNFL volume in women than men. Similarly, in the study by Ooto et al.,[11] mean mRNFL thickness was 31.6 +/- 3.07 μm for men and 32.8 +/- 3.7 μm (p = 0.006) for women. Despite being statistically significant, the differences between both sexes in the thickness of individual retinal layers are minimal and bellow the resolution and the variability of Spectralis OCT.

### Study limitations

The main limitations of our study were that participants were all European descent such that data collected may not be translatable to other ethnicities. Also its cross-sectional nature means that the significant changes with age detected require longitudinal studies to properly calculate the rate of thinning/thickening of the different retinal layers with age. Since Spectralis OCT resolution nowadays is not able to capture differences in the thickening bellow 4 μm, the thickness changes found in this study will need to be ascertain in future with more precise technology. And finally, the reader should be cautious when translating this results to clinical practice since the R values found in this study suggested that the thickness changes with age are statistically significant but minimal.

In conclusion, our findings indicate thickness changes associated with age in the majority of the retinal layers examined. No significant impacts of age were detected on mRNFL, INL or RPE thicknesses measured with the Spectralis OCT. Our data also confirm the greater overall thickness of the retina in men than women.

## Supporting information

S1 Dataset Results of the eccentricity analysis of retinal layers thickness. (CSV)
